# Maf1 ameliorates cardiac hypertrophy by inhibiting RNA polymerase III through ERK1/2

**DOI:** 10.7150/thno.33006

**Published:** 2019-09-25

**Authors:** Yu Sun, Cong Chen, Ruicong Xue, Yan Wang, Bin Dong, Jiayong Li, Chen Chen, Jingzhou Jiang, Wendong Fan, Zhuomin Liang, Huiling Huang, Rong Fang, Gang Dai, Youchen Yan, Tiqun Yang, Xiangxue Li, Zhan-Peng Huang, Yugang Dong, Chen Liu

**Affiliations:** 1 Department of Cardiology, the First Affiliated Hospital of Sun Yat-Sen University, Guangzhou, Guangdong, China, 510080; 2 Department of Cardiology, the Second People's Hospital of Guangdong Province, Guangzhou, Guangdong, China, 510080; 3 NHC Key Laboratory of Assisted Circulation (Sun Yat-sen University), Guangzhou, China, 510080; 4 Division of Cardiology, Department of Medicine, the University of Hong Kong Shenzhen Hospital, Shenzhen, Guangdong, China, 518000; 5 Department of Cardiology, Center for Translational Medicine, The First Affiliated Hospital, Sun Yat-sen University, Guangzhou, China.

**Keywords:** Maf1, cardiac hypertrophy, RNA polymerase III, ERK1/2

## Abstract

**Rationale**: An imbalance between protein synthesis and degradation is one of the mechanisms of cardiac hypertrophy. Increased transcription in cardiomyocytes can lead to excessive protein synthesis and cardiac hypertrophy. Maf1 is an RNA polymerase III (RNA pol III) inhibitor that plays a pivotal role in regulating transcription. However, whether Maf1 regulates of cardiac hypertrophy remains unclear.

**Methods**: Cardiac hypertrophy was induced *in vivo* by thoracic aortic banding (AB) surgery. Both the *in vivo* and *in vitro* gain- and loss-of-function experiments by Maf1 knockout (KO) mice and adenoviral transfection were used to verify the role of Maf1 in cardiac hypertrophy. RNA pol III and ERK1/2 inhibitor were utilized to identify the effects of RNA pol III and ERK1/2. The possible interaction between Maf1 and ERK1/2 was clarified by immunoprecipitation (IP) analysis.

**Results**: Four weeks after surgery, Maf1 KO mice exhibited significantly exacerbated AB-induced cardiac hypertrophy characterized by increased heart size, cardiomyocyte surface area, and atrial natriuretic peptide (ANP) expression and by exacerbated pulmonary edema. Also, the deficiency of Maf1 causes more severe cardiac dilation and dysfunction than wild type (WT) mice after pressure overload. In contrast, compared with adenoviral-GFP injected mice, mice injected with adenoviral-Maf1 showed significantly ameliorated AB-induced cardiac hypertrophy. *In vitro* study has demonstrated that Maf1 could significantly block phenylephrine (PE)-induced cardiomyocyte hypertrophy by inhibiting RNA pol III transcription. However, application of an RNA pol III inhibitor markedly improved Maf1 knockdown-promoted cardiac hypertrophy. Moreover, ERK1/2 was identified as a regulator of RNA pol III, and ERK1/2 inhibition by U0126 significantly repressed Maf1 knockdown-promoted cardiac hypertrophy accompanied by suppressed RNA pol III transcription. Additionally, IP analysis demonstrated that Maf1 could directly bind ERK1/2, suggesting Maf1 could interact with ERK1/2 and then inhibit RNA pol III transcription so as to attenuate the development of cardiac hypertrophy.

**Conclusions**: Maf1 ameliorates PE- and AB-induced cardiac hypertrophy by inhibiting RNA pol III transcription via ERK1/2 signaling suppression.

## 1. Introduction

Cardiac hypertrophy, an adaptive response of the myocardium to a variety of physiological and pathological stimuli, is regarded as a leading cause of heart failure. At the molecular level, cardiac hypertrophy is characterized by the re-expression of the fetal gene program and an imbalance between protein synthesis and degradation 1-3. As previously shown, prolonged external hypertrophic stimuli markedly activate the process of gene transcription, resulting in excessive protein accumulation in cardiomyocytes and leading to the development of cardiac hypertrophy 4. Pathological hypertrophy has been associated with abnormalities leading to the enhancement of the IGF1-phosphoinositide 3-kinase (PI3K)-Akt pathway, mitogen activated protein kinases (MAPKs), and Gαq signaling. Current therapies for cardiac hypertrophy include drug therapy, implantation of devices and surgery. Cardiac hypertrophy medications include angiotensin-converting enzyme inhibitor (ACEI), diuretics, β-blockers, ARBs, hydrolazine, and nitrates. These medications can regulate cardiac hypertrophy by regulating renin-angiotensin-aldosterone system (RAAS), β-AR and volume load. However, current drug treatments usually delay cardiac hypertrophy progression rather than regressing it. Therefore, therapeutic strategies that inhibit gene transcription or protein synthesis processes are promising for the treatment of cardiac hypertrophy 5-9.

Over the last decade, many studies have demonstrated that RNA polymerase (RNA pol) I, II, and III, which catalyze DNA-dependent RNA synthesis, are responsible for the transcription of the eukaryotic genome 10. RNA pol III, which mediates the production of small, untranslated RNA molecules, such as tRNA, 7SL RNA, snRNA and 5S rRNA, is essential for and involved in many fundamental processes, such as protein and ribosome biogenesis and RNA processing. RNA pol III activity is a critical determinant of cell growth and has been shown to be activated during myocardial hypertrophy 11, 12. Previous studies have demonstrated that the yeast RNA pol III transcription machinery consists of three transcription initiation factors, transcription factor IIIA (TFIIIA), transcription factor IIIB (TFIIIB), and transcription factor IIIC (TFIIIC). The functions of TFIIIB are pivotal to this transcription machinery, because TFIIIB can directly recruit RNA pol III to the transcriptional start site 13. Therefore, regulation of transcription by RNA pol III plays an important role in the regulation of cardiac hypertrophy.

Maf1, a protein that is conserved from yeast to humans, was first discovered in *Saccharomyces cerevisiae* and has since been identified in a variety of organisms; Maf1 is capable of inhibiting RNA polymerase III, thereby reducing protein synthesis by inhibiting transcription 14. Under nonstress conditions, Maf1 is phosphorylated and largely localized in the cytoplasm. Under stress conditions, Maf1 is rapidly dephosphorylated and accumulated in the nucleus 15, 16. Studies have shown that Maf1 represses pol III in response to stress conditions, such as DNA damage, oxidative stress, stationary phase growth, rapamycin or chlorpromazine treatment and secretory pathway inhibition 17, 18. Altogether, stress leads to Maf1 dephosphorylation and nuclear import, which enables Maf1 to bind pol III and prevent its interaction with TFIIIB and promoters, thereby inhibiting transcription 19, 20. Maf1 also interacts with Brf1, a subunit of TFIIIB that resembles the pol II initiation factor TFIIB 19, to repress transcription. Similar results have also been obtained in studies with mammal cells 21.

Although Maf1 is a key regulatory factor of RNA pol III and might affect protein synthesis, whether Maf1 can regulate cardiac hypertrophy remains poorly understood. Therefore, to explore the effect of Maf1 on cardiac hypertrophy and the underlying mechanism, we studied the role of Maf1 in the process of cardiac hypertrophy both* in vivo* and *in vitro*.

## 2. Methods

### 2.1 Reagents

Monoclonal antibodies against phospho-ERK1/2 (#8544S), ERK1/2 (#4695S), phospho-AKT (#9611S), AKT (#4685S), U0126 and cell lysis buffer were purchased from Cell Signaling Technology. Anti-Maf1 antibody (sc-365312, sc-515614) was obtained from Santa Cruz Biotechnology. DMEM/F12 and newborn bovine serum (NBS) were purchased from HyClone. Collagenase and trypsin were purchased from Gibco. Phenylephrine (PE) was purchased from Tokyo Chemical Industry. TRIzol was obtained from Sigma. RNA pol III inhibitor was purchased from Merck. Puromycin and anti-puromycin antibody were obtained from Sigma.

### 2.2 Neonatal rat myocyte and cardiac fibroblasts culture and recombinant adenovirus and siRNA transfection

Primary cultures of neonatal rat ventricular myocytes (NRVMs) were prepared under aseptic conditions from 1-to 3-day-old Sprague-Dawley rats obtained from the Experimental Animal Facility of Sun Yat-sen University. First, the hearts were excised from rats, and the adipose, atrial and blood vessel tissue were separated from the ventricles, which were placed in D-Hanks solution. Then, the ventricle tissue was cut into 2mm^3^ pieces and dissociated in 0.125% trypsin at 37°C for 5 minutes. The cell suspensions were separated and immersed again in D-Hanks solution three times, redigested in 0.05% collagenase at 37℃ for 2.5 hours, and neutralized with an equal volume of DMEM containing 10% NBS. Cell suspensions were collected. All suspensions were collected by centrifugation at 510 rpm for 8 minutes. The suspensions were separated and dipped in D-Hanks solution. After this step was repeated twice, the isolated cells were resuspended in DMEM containing 10% NBS and preplated for 1 hour to remove fibroblasts. Unattached NRVMs in DMEM containing 10% NBS and 1% 5-BrdU were plated at a density of 1×10^6^ cells/well in 6-well plates and incubated in a humidified atmosphere of 95% air/5% CO2 at 37℃. After 36 hours in culture, cardiomyocytes were handled accordingly. The adherent fibroblasts are isolated and passed to the second generation 22-24. Thirty-six hours after seeding, recombinant adenovirus was added (multiplicity of infection=5) to media containing 1% NBS to induce Maf1 overexpression. Similarly, a lipofectamine RNAiMAX transfection reagent (Invitrogen) was used for small interfering RNA (siRNA, purchased from RiboBio) transfection twenty-four hours after seeding, as recommended by the manufacturer's instructions. The sequence of the siRNA targeting Maf1 (si-Maf1) was as follows: 5' CAGCCAGAAGUCACGAAUU dTdT 3'. Twelve hours after adenovirus or siRNA transfection, the cells were treated with serum-free media for twenty-four hours and then treated with 50 μM PE for different durations according to the experiment.

Adenovirus vectors harboring Maf1 (Ad-Maf1) were generated using the E1/E3-deleted adenovirus vector system, and adenovirus vectors harboring GFP (Ad-GFP) were used as controls. Both adenoviruses were purchased from SinoGenoMax Co., Ltd.

### 2.3 Experimental animals

All of the experimental protocols complied with the Guide for the Care and Use of Laboratory Animals published by the US National Institutes of Health and the Animal Care and Use Committees of Sun Yat-Sen University. Eight- to 10-week-old male C57BL/6J mice were anesthetized with sodium pentobarbital (75 mg/kg, IP) and were endotracheally intubated. Cardiac hypertrophy was induced by pressure overload. First, adenoviruses (1×10^9^ total particles, 10 μl) diluted in phosphate-buffered saline (PBS) were injected into the left ventricular (LV) cavity, and then, aortic banding (AB) or sham procedures were performed as previously described 25. Briefly, the left thorax of C57BL/6J mice was opened at the second intercostal space, and a 7-0 silk suture ligature was tied around the descending aorta against a 26-gauge needle. Then, the needle was quickly removed. The detail information of *in vivo* myocardial gene transfer was provided in the supplement methods. A similar surgery was performed in the sham-operated mice with the exception of AB. Details regarding the construction of Maf1 knockout (Maf1 KO) mice are in the supplemental methods.

### 2.4 Cell surface area measurements

Cardiomyocytes were visualized using a charge-coupled device camera (Olympus, Japan) and were analyzed using Image-Pro Plus 6.0. To measure the mean cell surface area, 50-100 cells from randomly selected fields in each group were measured using Image-Pro Plus 6.0 26.

### 2.5 Echocardiography

Four weeks after the AB or sham surgery, mice were placed in the supine position on a table and anesthetized with isoflurane (2%) that was vaporized in 100% O_2_ using a constant-flow ventilator. Echocardiography was performed using ultrasonography.

### 2.6 Cytoplasmic and nuclear protein extraction

Fresh heart tissues were separated into cytoplasmic and nuclear protein fractions using a protein extraction kit (BioVision Research). First, the fresh tissue was cut into small pieces; then, ice-cold PBS was added, and the tissues were homogenized in a tissue homogenizer. The sample was vortexed vigorously and incubated on ice for 10 minutes. Then, ice-cold Cytosol Extraction Buffer-B was added to the tube, which was vortexed and incubated on ice for 1 minute. Afterward, the tube was vortexed and centrifuged. Then, the supernatant (cytoplasmic extract) fraction was immediately transferred to a clean prechilled tube and placed on ice. The pellet (containing the nuclei) was resuspended in ice-cold Nuclear Extraction Buffer Mix. Then, the sample was vortexed and placed on ice. This step was repeated every 10 minutes for a total of 40 minutes. Subsequently, the tube was centrifuged, and the supernatant (nuclear extract) was immediately transferred to a clean prechilled tube.

### 2.7 Immunoblot analyses

Immunoblot analyses were performed using standard procedures as previously described 27. The membrane was blocked with blocking buffer (1×TBS, 0.1% Tween-20, 5% BSA) for 1 hour at room temperature and then incubated overnight at 4°C with anti-phosphorylated-ERK1/2 (1:3000 dilution), anti-total-ERK1/2 (1:3000 dilution), anti-phosphorylated-Akt (1:1000 dilution), anti-total-Akt (1:1000 dilution), anti-Vinculin (1:10000 dilution), anti-Maf1 (1:300 dilution), anti-puromycin (1:10000 dilution) or anti-GAPDH (1:10000 dilution) primary antibodies. Then, the membrane was washed with TBS-T and incubated with secondary antibodies (1:10000 dilution, Protein-tech Group, Wuhan, China) at 37°C for 1 hour. The immune complex was detected with an enhanced chemiluminescence system (Millipore, Massachusetts, USA) and exposed to X-ray film. Quantitative analysis was performed using Quantity One software.

### 2.8 Immunofluorescence staining

Cultured cardiomyocytes that were grown on chamber slides were washed three times with PBS, fixed with 4% paraformaldehyde and permeabilized with PBS containing 0.1% Triton X-100. Then, the cells were blocked with PBS containing 2% bovine serum albumin for 60 minutes and stained with different antibodies. Images of Maf1 staining with Troponin I and DAPI staining were acquired by confocal microscopy.

### 2.9 Real-time quantitative PCR and reverse transcription (RT-PCR)

Methods of RNA extraction, reverse transcription and real-time quantitative PCR have been previously described 27. The primers for Maf1, atrial natriuretic peptide (ANP), GAPDH, ARPP P0, tRNA^Leu^, 5SrRNA and Brf1 were purchased from Sangon Biotech (Shanghai) Co., Ltd. The following are the sequences of the primers used in the study:

ANP (rat), sense 5'-TGAGCCGAGACAGCAAACATC-3' and antisense 5'-AGGCCAGGAAGAGGAAGAAGC-3'.

ANP (mouse), sense 5'-ACCTGCACCACCTGGAGG-3' and antisense 5'-CCTTGGCTGTTATCTTCGGTACCG-3'.

GAPDH (rat), sense 5'-ACAGCAACAGGGTGGTGGAC-3' and antisense 5'-TTTGAGGGTGCAGCGAACTT-3'.

GAPDH (mouse), sense 5'-GTTGTCTCCTGCGACTTCAAC-3' and antisense 5'-GCTGTAGCCGTATTCATTGTCA-3'.

ARPP P0 (rat), sense 5'-GCACTGGAAGTCCAACTACTTC-3' and antisense 5'-TGAGGTCCTCCTTGGTGAACAC-3'.

ARPP P0 (mouse), sense 5'-CCAGCAGGTGTTTGACAACG-3' and antisense 5'-TCCAGAAAGCGAGAGTGCAG-3'.

Brf1 (rat), sense 5'-CCTGGACACAGCCTTCAACT-3' and antisense 5'-GGTCGAGGAGCATGTCAAGT-3'.

Brf1 (mouse), sense 5'-TTTACCAGCAATCCCCAGGC-3' and antisense 5'-AGCTGGCTGGACTTCTTGAC-3'.

tRNA^Leu^, sense 5'-GTCAGGATGGCCGAGTGGTCTAAGGCGCC-3' and antisense 5'-CCACGCCTCCATACGGAGACCAGAAGACCC-3'.

5SrRNA, sense 5'-GGCCATACCACCCTGAACGC-3' and antisense 5'-CAGCACCCGGTATTCCCAGG-3'.

### 2.10 Immunoprecipitation (IP)

IP was performed using monoclonal antibodies against ERK1/2 from CST and against Maf1 from Santa Cruz. The Pierce Classic IP Kit (26146) from Thermo Scientific was used following the manufacturer's instructions. Firstly, the cells were washed once with PBS, and ice-cold IP lysis buffer was added to the cells. Then, the lysate was transferred to a tube and centrifuged to pellet the cell debris. Meanwhile, 80 μl of control agarose resin slurry (40 μl of settled resin) was added to a spin column and centrifuged to remove the storage buffer. Then, 100 μl of 0.1 M sodium phosphate and 0.15 M sodium chloride; pH 7.2 solution was added to the column, which was centrifuged; the flow-through was discarded. A sample of lysate (1 mg) was added to the column containing the resin and incubated at 4ºC for 30 minutes to 1 hour with gentle end-over-end mixing. Then, 1 µg of affinity purified antibody was combined with the precleared cell lysate in a microcentrifuge tube and incubated overnight at 4ºC to form the immune complex. The antibody/lysate sample was subsequently added to protein A/G plus agarose in the spin column. The screw cap was attached, and the column was then incubated with gentle end-over-end mixing or shaking for 1 hour. After washing the resin several times, 50 μl of 2×nonreducing lane marker sample buffer was prepared, and DTT was added for a final concentration of 20 mM. The spin column containing the resin was placed in a new collection tube, and the 2×reducing sample buffer was added. With the column unplugged and in the collection tube, the samples were incubated at 100ºC for 5 minutes. The columns were then centrifuged to collect the eluate. The samples were allowed to cool to room temperature before applying to an SDS-PAGE gel.

### 2.11 Statistical analysis

All of the data are expressed as the mean±standard error of the mean (SEM). The differences between the means were evaluated using one-way or two-way ANOVA. Statistical significance was established at *p*<0.05. The statistical analyses were performed using SPSS 13.0 software.

## 3. Results

### 3.1 Maf1 might be involved in AB-induced cardiac hypertrophy

**Figure 1:**
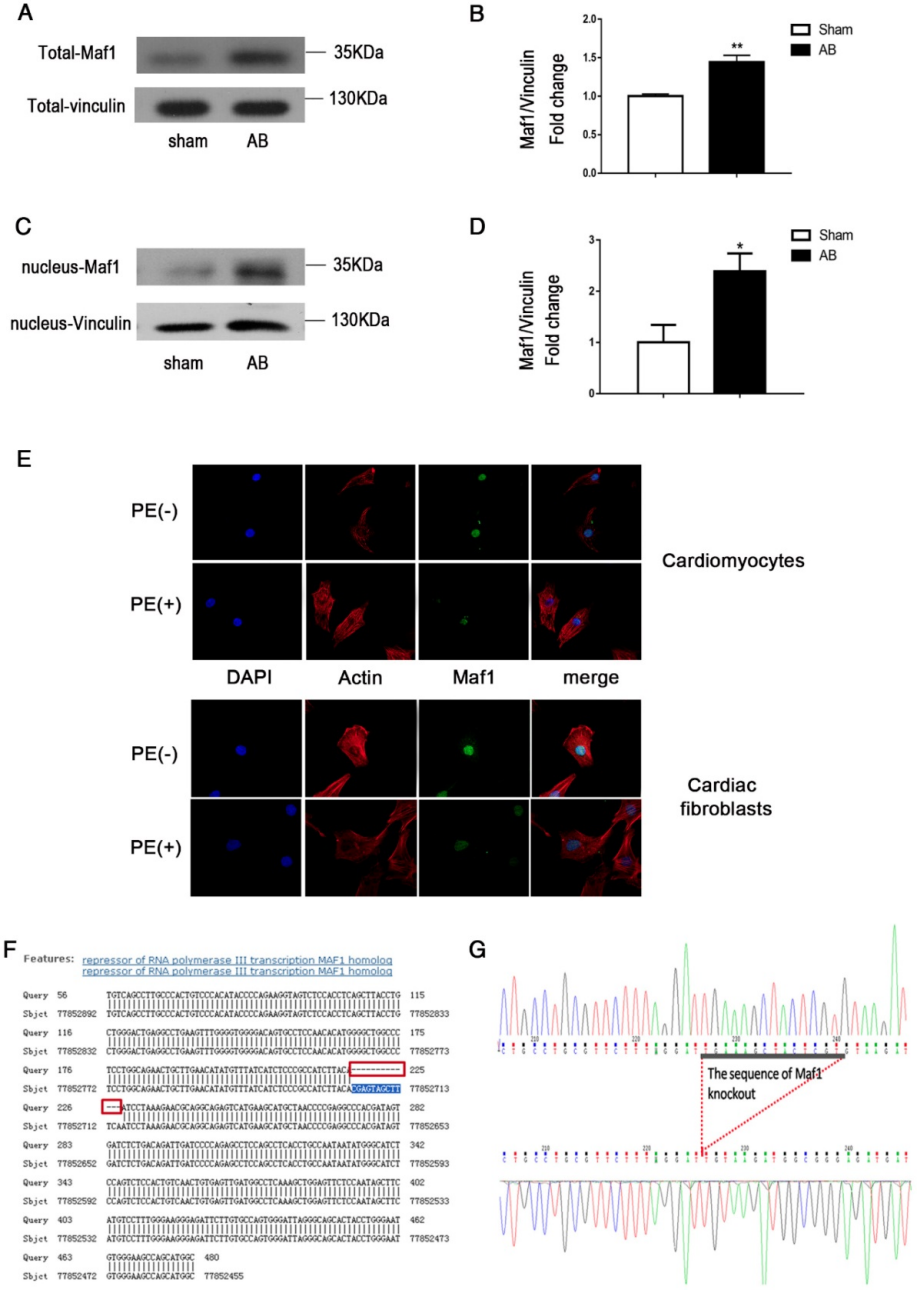
Effect of AB on Maf1 expression in cardiomyocytes and the genomic sequence of Maf1 knockout mice. The experiments in Figure 1A-D and Figure 1F-G were carried out in mice, while other experiments were carried out in primary cardiomyocytes and cardiac fibroblasts of SD rats. (A-B) Western blots showing total Maf1 and total vinculin expression in mouse hearts at 4 weeks after sham or AB surgery, and the quantitative analysis of those blots; n=6. (C-D) Western blots showing nuclear Maf1 and nuclear vinculin expression in mouse hearts at 4 weeks after sham or AB surgery, and the quantitative analysis of those blots. Vinculin was used as an internal control; n=3. (E) The cardiomyocytes were treated with 50 μM PE for 24 hours. Cardiomyocytes were stained with troponin I, the nuclei were stained with DAPI, and Maf1 was stained with a special antibody. (F-G) Maf1 deficiency was identified through genetic sequencing, and 13 bases of Maf1 were deleted. *p<0.05 versus the corresponding control group. **p<0.01 versus the corresponding control group.

To determine whether Maf1 participates in the cardiac hypertrophy process, we examined the changes in total and nuclear Maf1 expression in pressure overload-induced cardiac hypertrophy. We evaluated the expression of Maf1 *in vivo* using mouse hearts 4 weeks after AB or sham surgery. As shown in Figure 1A-D, total expression of Maf1 protein were increased about 44% and nuclear expression of Maf1 protein were increased about 2.4-fold 4 weeks after pressure overload in mice compared with the sham group.

Furthermore, as shown in Figure 1E, PE treatment altered the expression of Maf1 in cardiomyocytes and cardiac fibroblasts. When PE was added, the fluorescence intensity of Maf1 was significantly decreased both in nuclei and cytoplasm, which suggested that the expression alteration of Maf1 in PE-induced cardiomyocyte hypertrophy might be responsible for the change in RNA pol III transcription. In addition, we have detected the total and nuclear protein expressions of Maf1 in cardiomyocytes treated with recombinant adenovirus. As shown in the Figure S1A-D, total expression of Maf1 protein was increased about 4-fold and nuclear expression of Maf1 protein was increased about 3-fold compared with the control group. These findings suggested that alterations in Maf1 expression may participate in the development of cardiac hypertrophy.

### 3.2 Knockout of Maf1 exacerbates AB-induced cardiac hypertrophy and cardiac dysfunction

**Figure 2:**
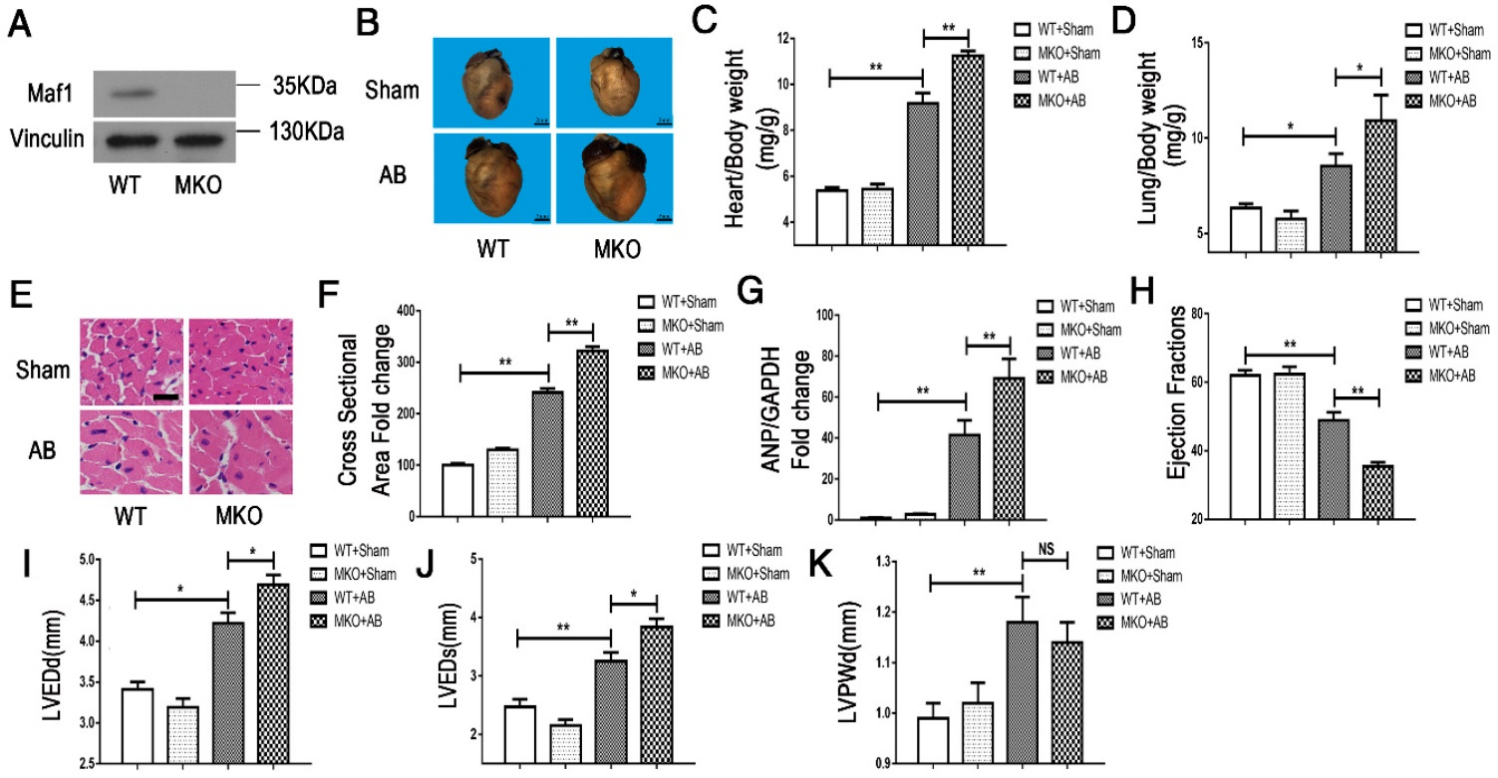
Effect of Maf1 knockout on AB-induced hypertrophy. The experiments were all carried out in mice. (A) Western blots showing Maf1 and vinculin expression in mouse hearts. Vinculin was used as an internal control. (B) Images of whole hearts from Maf1 knockout mice at 4 weeks after sham or AB surgery. (C-D) The heart or lung/body weight ratios were determined and showed that knockout of Maf1 could exacerbate AB-induced hypertrophy and pulmonary edema; WT+Sham n=7; MKO+Sham n=7; WT+AB n=8; MKO+AB n=6. (G) The effect of Maf1 knockout on ANP mRNA expression was determined by q-PCR, and GAPDH was used as an internal control; n=4-8. (E-F) Images of H&E-stained heart sections from mice at 4 weeks after sham or AB surgery; n=3. (H-K) The effects of Maf1 knockout on echocardiographic parameters (EF, LVEDd, LVEDs and LVPWd). *p<0.05 versus the corresponding control group. **p<0.01 versus the corresponding control group. NS indicates no significant difference versus the corresponding control group.

To investigate the effect of Maf1 on AB-induced cardiac hypertrophy *in vivo*, Maf1 knockout (Maf1 KO) mice were constructed. A transcription activator-like effector (TALE) nuclease targeting exon 3 of the Maf1 gene was designed using a target designer (*https://tale-nt.cac.cornell.edu/node/add/talen-old*). A small deletion was created in exon 3. As shown in Figure 1F-G, we identified Maf1 deficiency through genetic sequencing, which showed that 13 bases of Maf1 were deleted. Furthermore, Maf1 protein deficiency was detected by Western blot (Figure 2A). After confirming the successful knockout of Maf1, AB surgery was performed to induce cardiac hypertrophy by pressure overload. Before surgery, Maf1 KO mice has no significantly difference with wild-type (WT) mice, as determined by heart/body weight ratio, heart weight/tibia length ratio, lung weight/body weight, lung weight/tibia length ratio, ejection fraction (EF), left ventricular end-diastolic dimension (LVEDd), left ventricular end-systolic dimension (LVEDs) and end-diastolic left ventricular posterior wall thickness (LVPWd) (Table S1). At 4 weeks after AB surgery, the hearts of Maf1 KO mice were significantly larger than those of WT mice, exhibiting enhanced cardiac hypertrophy and pulmonary edema, as determined by increased heart size (about 30%) (Figure 2B), heart/body weight ratio (about 22%) (Figure 2C), heart weight/tibia length ratio (about 26%) (Figure S2A), lung weight/body weight (about 28%) (Figure 2D), lung weight/tibia length ratio (about 32%) (Figure S2B), cardiomyocyte surface area (about 33%) (Figure 2E-F), and expression of ANP (about 66%), a fetal gene (Figure 2G). In addition, Maf1 KO mice exhibited more severe cardiac dilation and dysfunction than WT mice after pressure overload. Compared with WT mice, Maf1-deficient mice showed a remarkably enlarged left ventricle and further impaired cardiac dysfunction, as determined by reduced EF (about 27%) (Figure 2H) and elevated LVEDd (about 11%) (Figure 2I) and LVEDs (about 18%) (Figure 2J). However, compared with WT mice, Maf1 KO mice did not exhibit a significant difference in LVPWd (Figure 2K) (Figure S4A). Therefore, these results suggested that knockout of Maf1 significantly exacerbated AB-induced cardiac hypertrophy and heart failure *in vivo*.

### 3.3 Upregulation of Maf1 attenuates AB-induced hypertrophy

**Figure 3:**
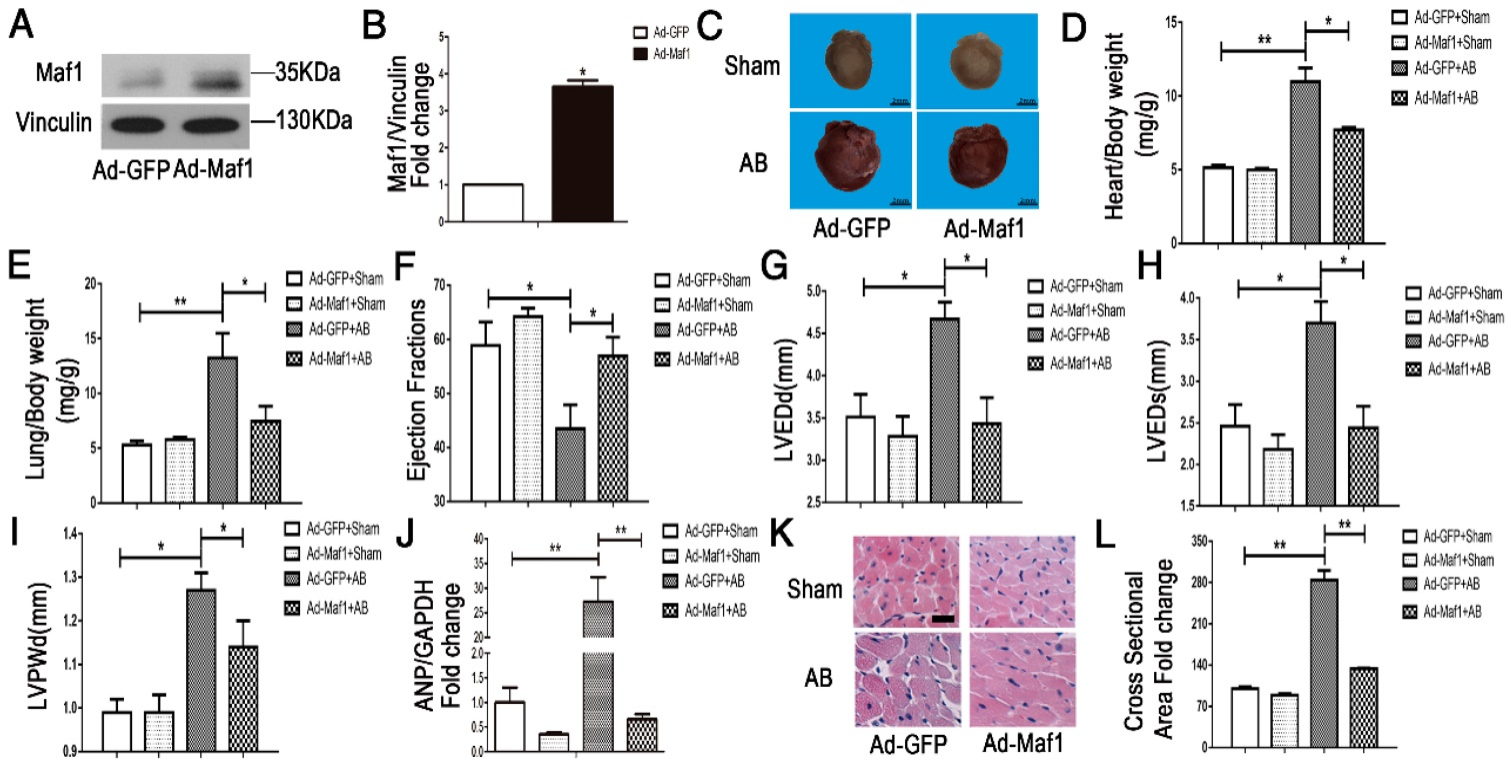
Effect of Maf1 upregulation on AB-induced hypertrophy. The experiments were all carried out in mice. (A-B) Western blots showing Maf1 and vinculin expression in mouse hearts, and the quantitative analysis of those blots. Vinculin was used as an internal control; n=3. (C) Images of whole hearts from Maf1-overexpressing mice 4 weeks after sham or AB surgery. (D-E) The heart or lung/body weight ratio was detected and showed that upregulation of Maf1 expression could attenuate AB-induced hypertrophy and pulmonary edema; Ad-GFP+Sham n=7; Ad-Maf1+Sham n=7; Ad-GFP +AB n=6; Ad-Maf1+AB n=5. (F-J) The effects of Maf1 upregulation on echocardiographic parameters (EF, LVEDd, LVEDs and LVPWd). (J) The effect of Maf1 upregulation on ANP mRNA expression was determined by q-PCR, and GAPDH was used as an internal control; n=4-5. (K-L) Images of H&E-stained heart sections from mice at 4 weeks after sham or AB surgery; n=3. *p<0.05 versus the corresponding control group. **p<0.01 versus the corresponding control group.

To study the effect of Maf1 overexpression on cardiac hypertrophy *in vivo*, adenoviral transfection was used to induce the overexpression of Maf1. Ad-maf1 (1×10^9^ total particles, 10 μl) diluted in PBS was injected into the LV cavity to upregulate Maf1 expression. The same amount of Ad-GFP was used as a control. This method could successfully transfect the mouse heart (Figure S3A). Four weeks after AB, the level of Maf1 was significantly increased about 3.5-fold in the hearts of the Ad-Maf1 group compared with of the Ad-GFP group (Figure 3A-B). Following overexpression of Maf1, the cardiomyocyte surface area and whole heart size were similar to those in the Ad-GFP mice after sham operation. Moreover, compared with the Ad-GFP-injected mice at 4 weeks after AB, the Maf1-overexpressing mice exhibited significantly attenuated cardiac hypertrophy and pulmonary edema, as shown by decreased heart size (about 30%) (Figure 3C), cardiomyocyte surface area (about 53%) (Figure 3K-L), heart/body weight ratio (about 30%) (Figure 3D), heart weight/tibia length ratio (about 19%) (Figure S3B), lung weight/body weight (about 43%) (Figure 3E) and lung weight/tibia length ratio (about 35%) (Figure S3C). As shown in Figure 3J, analysis of ANP expression showed that overexpression of Maf1 markedly attenuated the induction of this hypertrophic marker. Moreover, the echocardiographic results showed that the AB-induced increases in LVEDd (about 26%) (Figure 3G), LVEDs (about 34%) (Figure 3H), and LVPWd (about 10%) (Figure 3I) were markedly diminished by the upregulation of Maf1. In addition, the AB-induced decrease in EF was significantly improved by enhanced Maf1 expression (about 30%) (Figure 3F) (Figure S4B). Accordingly, these results suggest that upregulation of Maf1 significantly ameliorates AB-induced cardiac hypertrophy and heart failure *in vivo*.

### 3.4 Maf1 ameliorates cardiomyocyte hypertrophy *in vitro*

**Figure 4:**
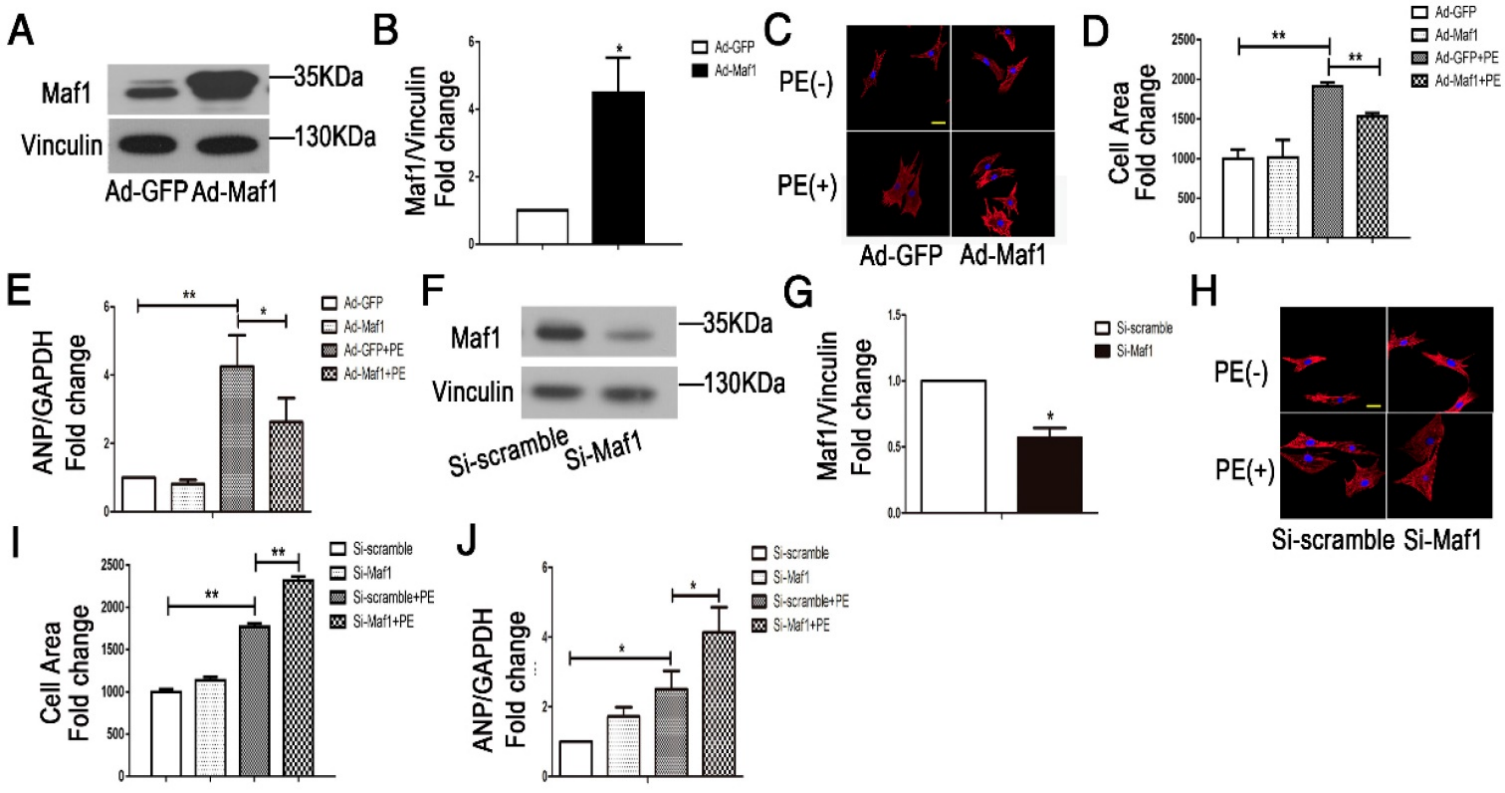
Effects of Maf1 knockdown and upregulation on PE-induced cardiomyocyte hypertrophy. The experiments were all carried out in primary cardiomyocytes of SD rats. The cardiomyocytes were treated with 50 μM PE for 24 hours. (A-B) Western blots showing the Maf1 overexpression in cardiomyocytes, and the quantitative analysis of those blots. Vinculin was used as an internal control; n=4. (C, H) Cardiomyocytes were stained for troponin I expression, and the nuclei were stained with DAPI. (D) The effect of Maf1 upregulation on the cell surface area; n=3. (E) The effect of Maf1 upregulation on ANP mRNA expression was determined by q-PCR, and GAPDH was used as an internal control; n=6. (F-G) Western blots showing Maf1 knockdown efficiency in cardiomyocytes, and the quantitative analysis of those blots. Vinculin was used as an internal control; n=4. (I) The effect of Maf1 knockdown on the cell surface area; n=3. (J) The effect of Maf1 knockdown on ANP mRNA expression was determined by q-PCR, and GAPDH was used as an internal control; n=4-5. *p<0.05 versus the corresponding control group. **p<0.01 versus the corresponding control group.

To further clarify the effect of Maf1 on cardiac hypertrophy, we examined the effect of Maf1 on PE-induced cardiomyocyte hypertrophy. We upregulated Maf1 expression in cardiomyocytes with recombinant adenovirus transfection and induced cardiomyocyte hypertrophy by PE treatment. The level of Maf1 gradually increased with an increasing multiplicity of infection (M.O.I.), overexpression of Maf1 by 4.5-fold was achieved by adenoviral transfection at an M.O.I. of 5 (Figure 4A-B). After PE treatment, the cardiomyocyte surface area was markedly increased by 2-fold compared with Ad-GFP group, while cardiomyocyte hypertrophy was significantly attenuated by Maf1 overexpression (about 20%) (Figure 4C-D). Moreover, the PE-induced increase in the level of ANP mRNA was also reversed in Ad-Maf1-treated cardiomyocytes (about 38%) (Figure 4E). Thus, consistent with the *in vivo* findings, overexpression of Maf1 inhibited cardiomyocyte hypertrophy *in vitro*.

To further confirm the role of Maf1, we downregulated Maf1 in cardiomyocytes by RNA silencing. Immunoblotting showed that Maf1 expression in siRNA-treated cardiomyocytes was decreased by 50% (Figure 4F-G). There was no difference in the cardiomyocyte surface area between the siRNA-transfected and control cardiomyocytes without PE treatment, but knockdown of Maf1 exacerbated PE-induced cell enlargement (about 31%) (Figure 4H-I). In contrast to the data from gain-of-function experiments, knockdown of Maf1 significantly elevated the level of ANP compared with that in the control group (about 65%) (Figure 4J). These results indicate that downregulation of Maf1 can exacerbate PE-induced cardiomyocyte hypertrophy *in vitro*.

### 3.5 RNA pol III transcription is involved in the inhibition of cardiac hypertrophy by Maf1

**Figure 5:**
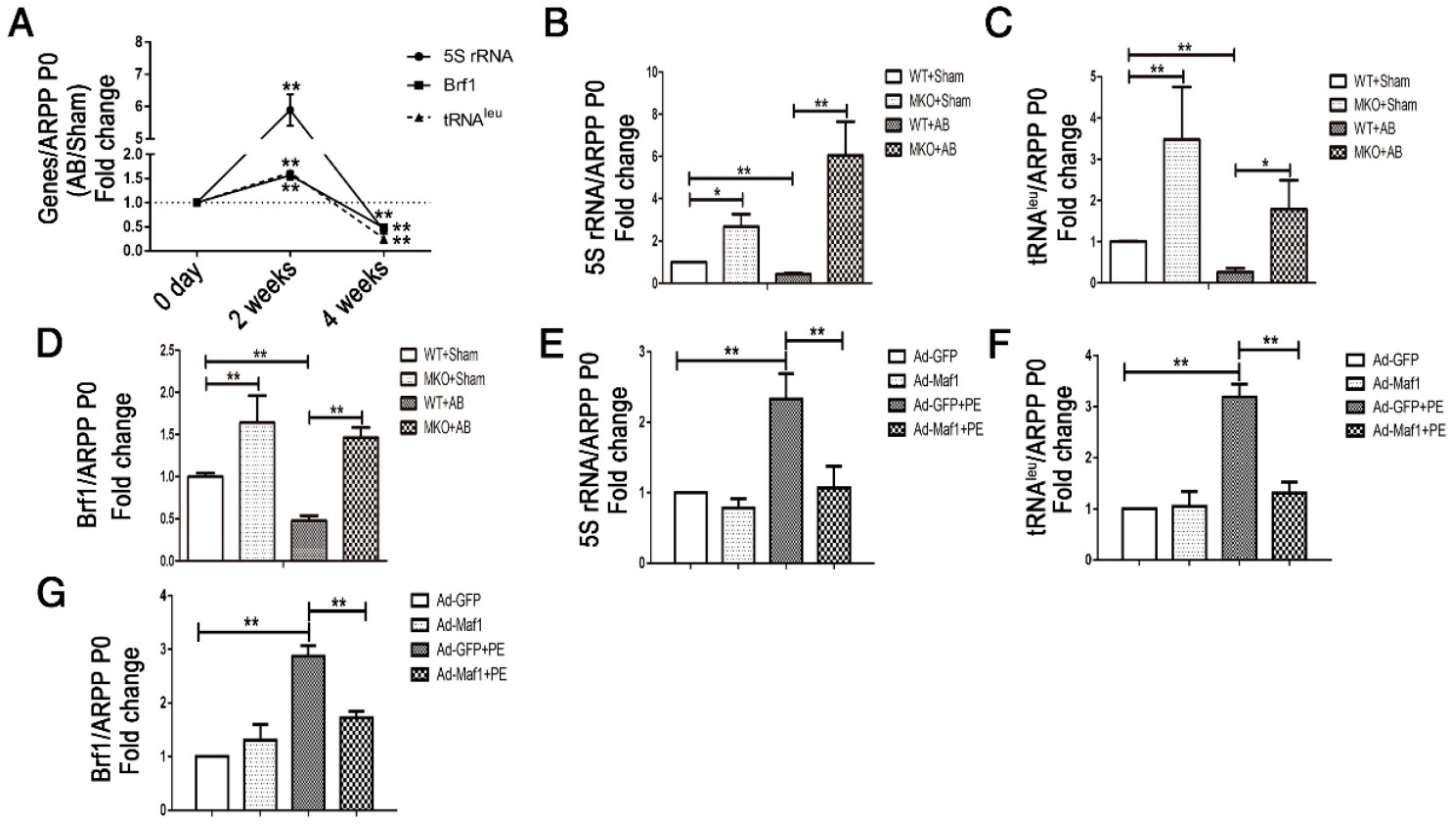
Maf1-mediated cardiac hypertrophy is related to RNA polymerase III transcription. The experiments in Figure 5A-D were carried out in mice, while other experiments were carried out in primary cardiomyocytes of SD rats. (A) Expression levels of the relevant products of RNA pol III transcription 5SrRNA and tRNA^Leu^ and the pol III subunit Brf1 were detected by q-PCR at 2 and 4 weeks after surgery, and ARPP P0 was used as an internal control; n=5-7.(B-D) Expression levels of the relevant products of RNA pol III transcription 5SrRNA and tRNA^Leu^ and the pol III subunit Brf1 were detected by q-PCR at 4 weeks after sham or AB surgery; n=4-6. (E-G) The cardiomyocytes were treated with 50 μM PE for 24 hours. Expression levels of the relevant products of RNA pol III transcription 5SrRNA and tRNA^Leu^ and the pol III subunit Brf1 were detected by q-PCR after PE treatment; n=6. *p<0.05 versus the corresponding control group. **p<0.01 versus the corresponding control group.

**Figure 6:**
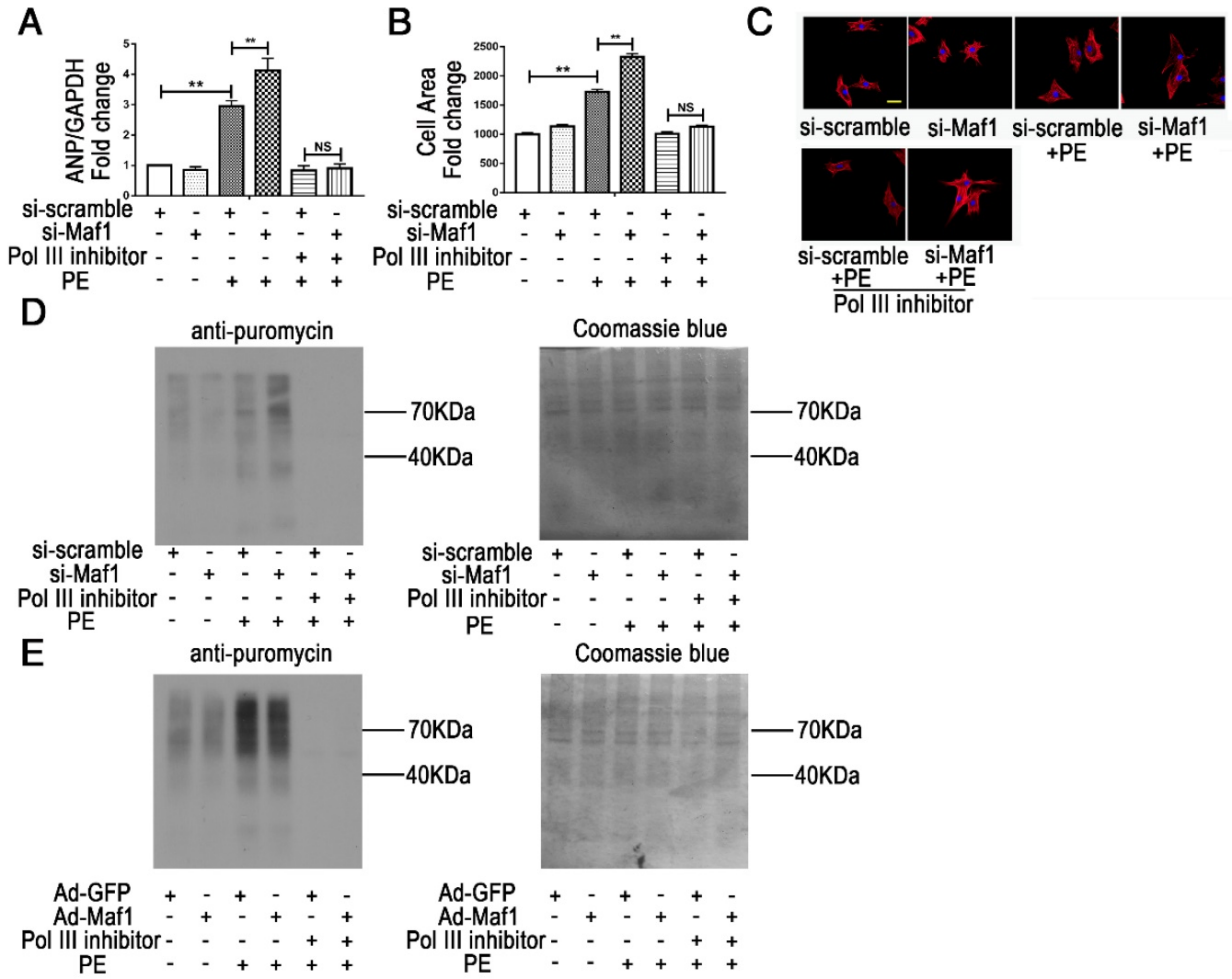
Maf1 ameliorates cardiac hypertrophy via negative regulation of RNA pol III transcription. The experiments were all carried out in primary cardiomyocytes of SD rats. The cardiomyocytes were treated with 50 μM PE for 24 hours. (A) The effects of Maf1 knockdown and pol III inhibition on ANP mRNA expression was determined by q-PCR, and GAPDH was used as an internal control; n=6. (B) The effects of Maf1 knockdown and pol III inhibition on cell surface areas; n=3. (C) Cardiomyocytes were stained for troponin I expression, and the nuclei were stained with DAPI. (D) Puromycin labeling to measure protein synthesis during si-Maf1-mediated cardiac hypertrophy and the effect of the pol III inhibitor on protein synthesis. (E) Puromycin labeling to measure protein synthesis in Ad-Maf1-regulated cardiac hypertrophy, and the effect of pol III inhibitor on protein synthesis. One micromolar puromycin was added in each well and incubated for 30 minutes. Cardiomyocytes were then collected and subjected to WB analysis. Coomassie blue was used as an internal control. **p<0.01 versus the corresponding control group. NS indicates no significant difference versus the corresponding control group.

RNA pol III plays a key role in protein synthesis by catalyzing the production of small, untranslated RNA molecules, such as tRNA and 5SrRNA, which are essential components of the biosynthetic apparatus 28. Brf1 is a subunit of TFIIIB that is essential for the initiation of RNA pol III transcription 29. Previous studies have shown that Maf1 is a key negative regulator of RNA pol III transcription in yeast and mammals. However, whether Maf1 inhibits cardiac hypertrophy by repressing pol III transcription is still unclear. Therefore, we detected the expression levels of the relevant products of RNA pol III transcription 5SrRNA and tRNA^Leu^ and the pol III subunit Brf1 by q-PCR. As shown in Figure 5A, 5SrRNA (about 6-fold), tRNA^Leu^ and Brf1 (about 1.5-fold) expression levels were significantly increased at 2 weeks but decreased at 4 weeks in the pressure overload group compared with the sham group. Importantly, knockout of Maf1 significantly increased these indicators by about 2-fold after either sham or AB surgery compared with those in WT mice (Figure 5B-D). Additionally, the *in vitro* study showed elevated levels of 5SrRNA, tRNA^Leu^ and Brf1 during the process of PE-induced cardiomyocyte hypertrophy. However, adenoviral overexpression of Maf1 markedly reduced the enhanced levels of the pol III transcription products and Brf1 (Figure 5E-G). To further explore the role of pol III in PE-induced cardiomyocyte hypertrophy, a pol III inhibitor was used. As shown in Figure 6A-C, the pol III inhibitor significantly blocked the pro-hypertrophic effect of Maf1 knockdown on PE-induced cardiomyocyte hypertrophy, in terms of decreased levels of ANP and the cardiomyocyte cell area compared with those in the corresponding control group. In addition to the changes in transcription, we applied puromycin labeling to measure protein synthesis *in vitro*. As shown in Figure 6D-E, we found that overexpression of Maf1 could inhibit protein synthesis while Maf1 knockdown could promote protein synthesis compared with the corresponding control groups. Meanwhile, these effects were markedly abolished by treatment with a pol III inhibitor. Taken together, these results indicate that Maf1 might inhibit cardiac hypertrophy through negative regulation of RNA pol III, which eventually leads to reduced protein synthesis.

### 3.6 Maf1 attenuates PE-induced cardiomyocyte hypertrophy through the ERK1/2 signaling pathway

**Figure 7:**
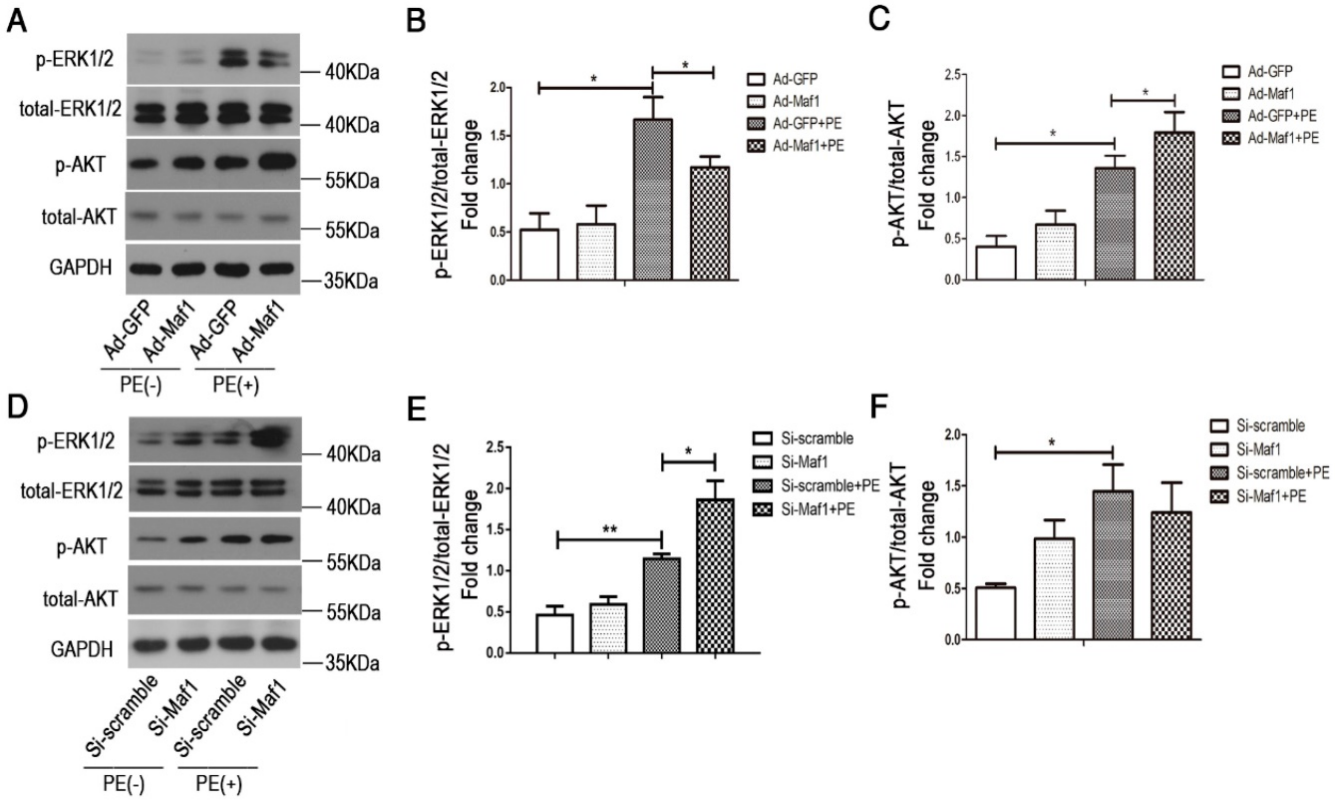
Effect of Maf1 expression changes on the ERK1/2 and AKT signaling pathways. The experiments were all carried out in primary cardiomyocytes of SD rats. The cardiomyocytes were treated with 50 μM PE for 30 minutes. (A-C) Western blots showing the effects of Maf1 upregulation on the ERK1/2 and AKT signaling pathways, and the quantitative analysis of those blots; n=5-6. (D-F) Western blots showing the effects of Maf1 knockdown on the ERK1/2 and AKT signaling pathways, and the quantitative analysis of those blots; n=4-6. *p<0.05 versus the corresponding control group.

**Figure 8:**
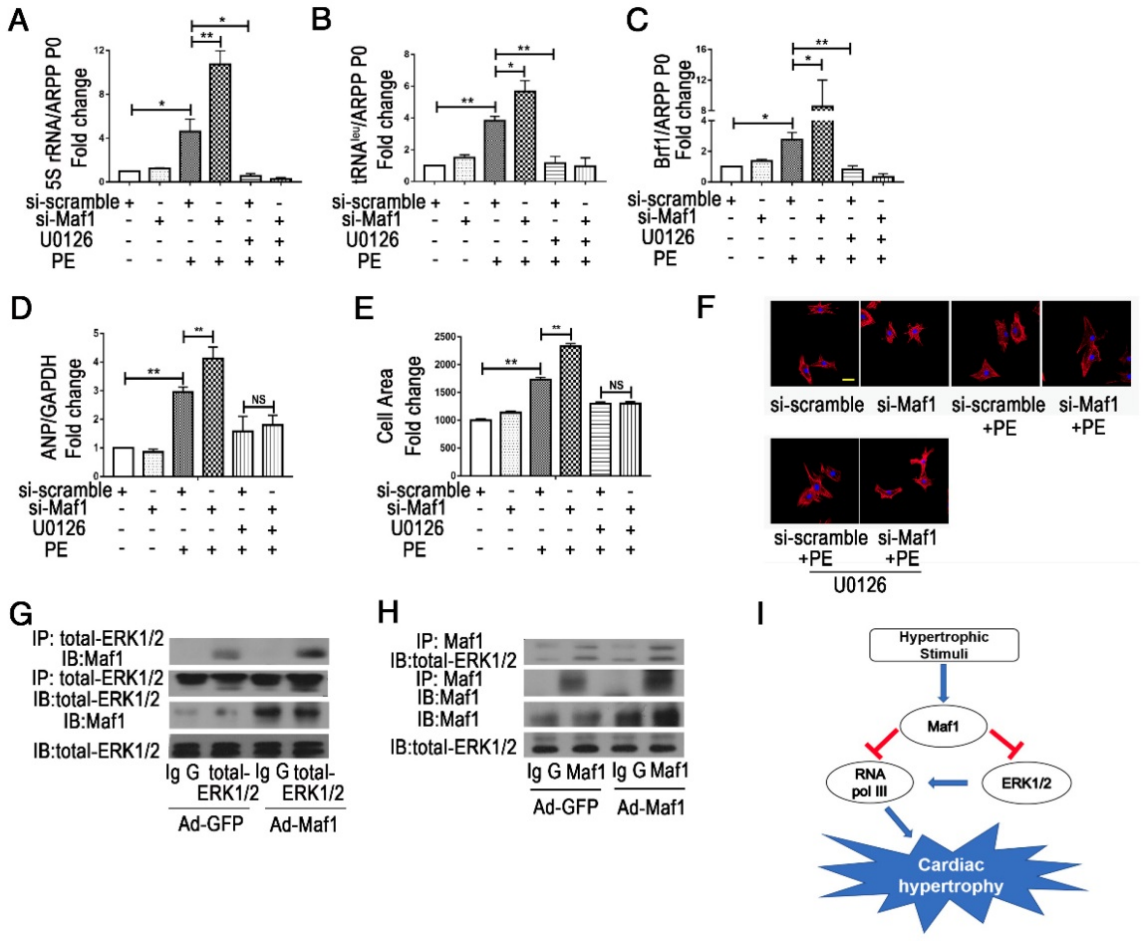
Maf1 ameliorates cardiac hypertrophy via ERK1/2 signaling pathways. The experiments were all carried out in primary cardiomyocytes of SD rats. The cardiomyocytes were treated with 50 μM PE for 24 hours. (A-C) The effects of Maf1 knockdown and U0126 treatment on expression levels of the relevant products of RNA pol III transcription 5SrRNA and tRNA^Leu^ and the pol III subunit Brf1 were detected by q-PCR, and ARPP P0 was used as an internal control; n=3-6. (D) The effects of Maf1 knockdown and U0126 treatment on ANP mRNA expression was determined by q-PCR, and GAPDH was used as an internal control; n=5-6. (E) The effects of Maf1 knockdown and U0126 treatment on the cell surface area; n=3. (F) Cardiomyocytes were stained for troponin I expression, and the nuclei were stained with DAPI. (G-H) IP analysis showed that Maf1 could directly bind ERK1/2. (I) The working model of this research. In this research, we have demonstrated that Maf1 could ameliorate cardiac hypertrophy via negative regulation of RNA pol III transcription by directly bindingERK1/2. *p<0.05 versus the corresponding control group. **p<0.01 versus the corresponding control group. NS indicates no significant difference versus the corresponding control group.

ERK1/2 and AKT signaling play important roles in the development of physiological and/or pathological cardiac hypertrophy. By evaluating pro-hypertrophic signaling pathways, including ERK1/2 and AKT, we found that ERK1/2 and AKT were activated in cardiac hypertrophy process. Our *in vitro* study also confirmed that upregulation of Maf1 repressed ERK1/2 signaling but activated AKT signaling (Figure 7A-C). Thus, we speculated that Maf1 overexpression negatively regulated cardiac hypertrophy mainly by blocking ERK1/2 signaling. To confirm these results, we investigated whether ERK1/2 and AKT signaling was altered in the cardiomyocytes transfected with Maf1 siRNA. As shown in Figure 7D-F, downregulation of Maf1 further facilitated the activation of ERK1/2 by PE treatment compared with that in the si-scramble group. However, downregulation of Maf1 did not significantly alter AKT phosphorylation compared with that in cardiomyocytes transfected with si-scramble. The results imply that the ERK1/2 signaling pathway plays a pivotal role in mediating the inhibitory effect of Maf1 on cardiac hypertrophy. Moreover, we have detected the alteration of ERK1/2 and AKT in Maf1 knockout mice. As shown in the Figure S5A-C, knockout of Maf1 facilitated the activation of ERK1/2 compared with WT mice treated with AB surgery. However, knockout of Maf1 did not significantly alter AKT phosphorylation. These results confirmed that the ERK1/2 signaling pathway could be one of the key downstream effectors of Maf1 in inhibiting cardiac hypertrophy. To elucidate whether Maf1 ameliorates cardiac hypertrophy by inhibiting ERK1/2, an ERK1/2 inhibitor (U0126) was used. As shown in Figure 8D-F, U0126 could significantly block the pro-hypertrophic effect of Maf1 knockdown. Moreover, U0126 markedly inhibited the expression of 5SrRNA, tRNA^Leu^ and the pol III subunit Brf1 under PE treatment (Figure 8A-C and Figure S6A-C). As the previous study by Goodfellow 11, 30 demonstrated, ERK1/2 could induce the activation of pol III transcription, acting as an upstream regulator of RNA pol III in cardiac hypertrophy. Thus, according to our findings, we speculated that Maf1 might regulate cardiac hypertrophy by modulating RNA pol III activity via the ERK1/2 signaling pathway.

However, whether Maf1 interacts with ERK1/2 remains unknown. To explore the potential interaction, IP analysis was performed. As shown in Figure 8G-H, Maf1 and ERK1/2 could directly interact with each other, and the effect was enhanced by overexpression of Maf1. The interaction between Maf1 and ERK1/2 implies that Maf1 might regulate cardiac hypertrophy through RNA pol III inhibition by directly binding ERK1/2.

## 4. Discussion

In the present study, we investigated the role of Maf1 in AB- and PE-induced cardiac hypertrophy and demonstrated that Maf1 could block cardiac hypertrophy *in vivo* and *in vitro.* The data show that Maf1 ameliorates cardiac hypertrophy by inhibiting pol III transcription via direct regulation of ERK1/2.

It is well known that enhanced protein synthesis is a hallmark of hypertrophy 1, 2, 31. Previous studies have shown that pol I transcribes the 28S, 18S, and 5.8S rRNAs as a single large precursor RNA, while pol III is responsible for the production of multiple transcripts that include small, untranslated RNA molecules, such as tRNA and 5S rRNA. These transcription processes account for greater than 80% of total RNA synthesis and, thus, coordinate the regulation of pol I and pol III transcription, which is vital for biosynthesis in cell growth 10, 29. Transcription by pol III is known to be tightly regulated in cardiac hypertrophy, it is not only induced during cardiomyocyte hypertrophy but also in the normal adult mouse heart 11, 30. Thus, identifying the molecules that target RNA pol III is desirable for regulating cardiac hypertrophy.

Maf1 is widely known as a key negative regulator of RNA pol III, and has been verified from yeast to humans 17, 18, 32. Previous studies have found that Maf1 KO mice are leaner than their WT counterparts by reducing food intake and increasing metabolic inefficiency at 24 weeks of age. Furthermore, it also reported that Maf1 knockout mice that are homozygous for the targeted mutation are viable and fertile, but produce fewer pups and have fewer birth events 19, 33. This finding is consistent with our research. However, whether Maf1 participates in cardiac hypertrophy process remains unclear. In the present study, we found that total and nuclear expression of Maf1 was significantly upregulated in AB-induced cardiac hypertrophy, suggesting that changes in Maf1 might play a role in the development of hypertrophy. To further clarify the relationship between Maf1 and cardiac hypertrophy, we first conducted gain- and loss-of-function studies with Maf1 knockout mice and by adenoviral overexpression of Maf1 in WT mice. In our present research, we found that Maf1 KO mice were not significantly different than WT mice before surgery, as determined by heart/body weight ratio, heart weight/tibia length ratio, lung weight/body weight, lung weight/tibia length ratio, EF, LVEDd, LVEDs and LVPWd. This phenotype indicated that knocking out of Maf1 does not affect cardiac hypertrophy in nonstress conditions. Furthermore, the findings demonstrated that Maf1 knockout excessively promoted cardiac hypertrophy and heart failure, which were characterized by higher heart/body weight ratio, heart weight/tibia length ratio, cardiomyocyte surface area, and ANP expression and by more severe cardiac dilation and dysfunction than those in WT mice after pressure overload. Meanwhile, adenoviral overexpression of Maf1 could ameliorate cardiac hypertrophy and heart failure. Moreover, our *in vitro* research confirmed the cardioprotective effect of Maf1 on PE-induced cardiomyocyte hypertrophy. Thus, both the *in vivo* and *in vitro* gain- and loss-of-function experiments verified that Maf1 could ameliorate cardiac hypertrophy and cardiac dysfunction. Taken together, we discovered for the first time that Maf1 has an inhibitory effect on the development of cardiac hypertrophy.

It is well established that hypertrophic growth is accompanied by increased RNA pol III transcription 11, 30. Although Maf1 is a key negative regulatory molecule of RNA pol III, whether Maf1 regulates cardiac hypertrophy through pol III remains unknown. Therefore, the relevant pol III products, 5SrRNA and tRNA^Leu^, and Brf1, the subunit of RNA pol III, were detected to evaluate the effects of Maf1 in our study. In accordance with a previous study 11, 30, the pol III transcripts were not only markedly elevated in PE-induced cardiomyocyte hypertrophy but also in mice after pressure overload. Meanwhile, significant increases in the levels of the pol III products were observed in mice with a genetic deficiency in Maf1 and accompanied by more severe cardiac hypertrophy and dysfunction after AB, indicating that loss of Maf1 leads to exacerbated heart failure that is correlated with elevated RNA pol III transcription. In addition, the gain-of-function experiments using recombinant adenoviruses confirmed the negative effect of Maf1 on pol III transcription in the process of cardiac hypertrophy. Importantly, the pharmacological blockade of pol III transcription markedly rescued the Maf1 knockdown-exacerbated cardiomyocyte hypertrophy, showing that Maf1 inhibits cardiac hypertrophy by negatively regulating RNA pol III transcription. Moreover, treatment with a pol III inhibitor significantly blocked the pro-hypertrophy effect of si-Maf1 by inhibiting protein synthesis, suggesting that the reduction of transcription by Maf1 leads to protein synthesis inhibition. Altogether, these results uncovered the role of pol III transcription in the regulation of cardiomyocyte hypertrophy by Maf1. Based on the effect of Maf1 on RNA pol III transcription, we demonstrated that Maf1 might repress cardiac hypertrophy by suppressing RNA pol III.

ERK1/2, the key component of MAPK signaling pathways, can regulate pol III transcription in cardiomyocytes and is critical in mediating the development of cardiac hypertrophy 11, 34. In a previous study, Goodfellow demonstrated that ERK signaling could induce Brf1 expression during cardiomyocyte hypertrophy or in the absence of hypertrophic stimuli 11, 30. Therefore, to further explore whether the ERK1/2 signaling pathway plays a critical role in Maf1-regulated cardiac hypertrophy, we detected the changes in the ERK1/2 signaling pathway in AB or PE-induced cardiac hypertrophy. In our present study, both *in vivo* and *in vitro* experiments confirmed that Maf1 inhibits ERK1/2 signaling and suggested that Maf1 functions as an antihypertrophic effector, mainly by inhibiting ERK1/2. However, 4 weeks after AB, we have not detected the induced phosphorylation of ERK1/2 and AKT signaling *in vivo* experiment. We speculate that this may be related to the time of detection. Furthermore, although lots of studies have demonstrated that Maf1 directly combined with RNA pol III 14, 16, 17, whether Maf1 could regulate pol III via ERK1/2 remains unclear. Hence, to further elucidate how Maf1 regulates cardiac hypertrophy by pol III and ERK1/2, we blocked pol III transcription and the ERK1/2 signaling pathway using an RNA pol III inhibitor and an ERK1/2 inhibitor (U0126), in PE-induced cardiomyocyte hypertrophy, respectively. We found that both RNA pol III inhibitor and U0126 inhibited the expression of 5SrRNA, tRNA^Leu^ and Brf1 and improved Maf1 knockdown-promoted cardiac hypertrophy. Our results are consistent with the previous study by Goodfellow 11, 30 that RNA pol III participates in cardiac hypertrophy as a downstream target of ERK1/2. Thus, we further investigated whether Maf1 could directly or indirectly interact with ERK1/2 to regulate PE-induced cardiomyocyte hypertrophy. Coimmunoprecipitation demonstrated that Maf1 could directly bind ERK1/2, suggesting that ERK1/2 might be a core target in the Maf1-mediated inhibitory effects on RNA pol III and cardiac hypertrophy. This result is the first evidence that Maf1 could directly bind ERK1/2 and regulate cardiac hypertrophy. It suggests that Maf1/ERK/pol III axis may be an important mechanism for Maf1 in regulating pol III and is an important supplement to the mechanism of inhibiting pol III transcription by direct interaction between Maf1 and pol III in the context of cardiac hypertrophy. In conclusion, our *in vivo* and *in vitro* studies reveal that Maf1 can directly bind ERK1/2 and ameliorate cardiac hypertrophy via negative regulation of RNA pol III transcription, thus providing a new potential clinical treatment target for cardiac hypertrophy.

## Supplementary Material

Supplementary figures and tables.

## Abbreviations

AB: aortic banding
PE: phenylephrine
RAAS: renin-angiotensin-aldosterone system
ACEI: angiotensin-converting enzyme inhibitor
MAPKs: mitogen activated protein kinases
KO: knockout
WT: wild type
IP: immunoprecipitation
ANP: atrial natriuretic peptide
pol III: polymerase III
TFIIIA: transcription factor IIIA
TFIIIB: transcription factor IIIB
TFIIIC: transcription factor IIIC
NBS: newborn bovine serum
LVEDd: left ventricular end-diastolic dimension
LVEDs: left ventricular end-systolic dimension
LVPWd: left ventricular posterior wall, diastolic
