# Supplementary material for: Maf1 ameliorates cardiac hypertrophy by inhibiting RNA polymerase III through ERK1/2
Source: Theranostics. 2019 Sep 25;9(24):7268–81. doi: 10.7150/thno.33006 (PMC6831308; doi:10.7150/thno.33006)
Supplement: Supplementary file 1 — Supplementary figures and tables. [file thnov09p7268s1.pdf]

Supplement Table1

	WT (8 weeks)	MKO (8 weeks)
**Number**	n=6	n=6
**HW/BW(mg/g)**	5.04±0.15	5.13±0.09
**LW/BW(mg/g)**	5.09±0.13	5.75±0.43
**HW/TL(mg/mm)**	6.91±0.19	7.07±0.15
**LW/TL(mg/mm)**	6.98±0.15	7.93±0.61
**LVEDd(mm)**	3.02±0.11	3.10±0.12
**LVEDs(mm)**	2.06±0.11	2.17±0.10
**LVPWd(mm)**	0.78±0.02	0.81±0.03
**EF%**	61.67±2.48	56.38±2.48

Supplement Table1. The baseline data of WT and MKO mice was determined before surgery.

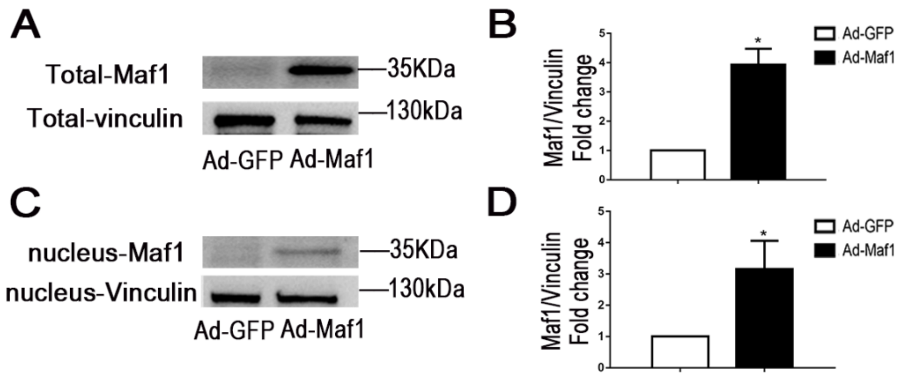

Supplement Figure 1. The total and nuclear Maf1 levels in cardiomyocytes treated with recombinant adenovirus. The experiments were carried out in primary cardiomyocytes of SD rats. (A-B) Western blots showing total Maf1 and total vinculin expression in cardiomyocytes, and the quantitative analysis of those blots. (C-D) Western blots showing nuclear Maf1 and nuclear vinculin expression in cardiomyocytes, and the quantitative analysis of those blots. n=3. *p<0.05 versus the corresponding control group.

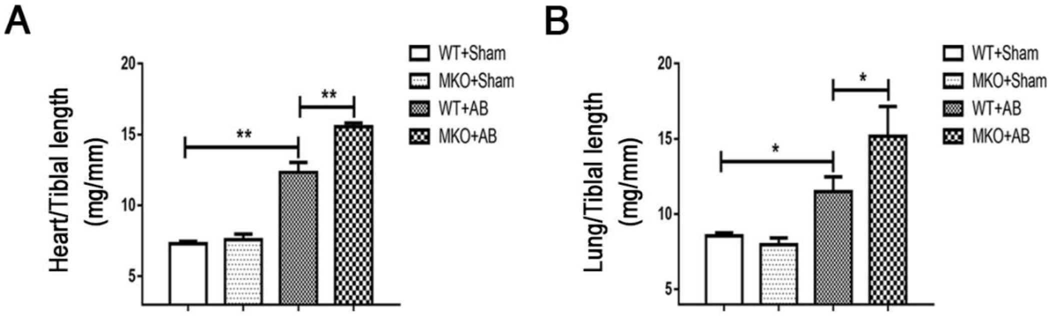

Supplement Figure 2. (A-B) The heart or lung/tibial length ratio was detected and showed that knockout of Maf1 could exacerbate AB-induced hypertrophy and pulmonary edema;

n=6-8. *$p<0.05$ versus the corresponding control group. **$p<0.01$ versus the corresponding control group.

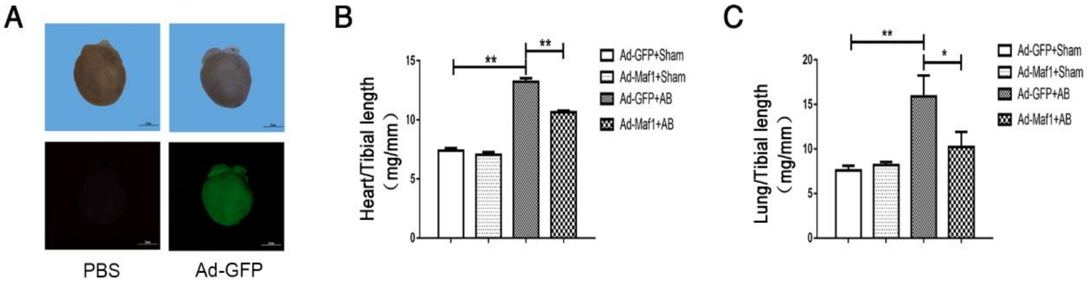

Supplement Figure 3. (A) Adenoviruses injected into the LV cavity could successfully transfect the mice heart. (B-C) The heart or lung/tibial length ratio was detected and showed that upregulation of Maf1 could attenuate AB-induced hypertrophy and pulmonary edema; n=5-7. *$p<0.05$ versus the corresponding control group. **$p<0.01$ versus the corresponding control group.

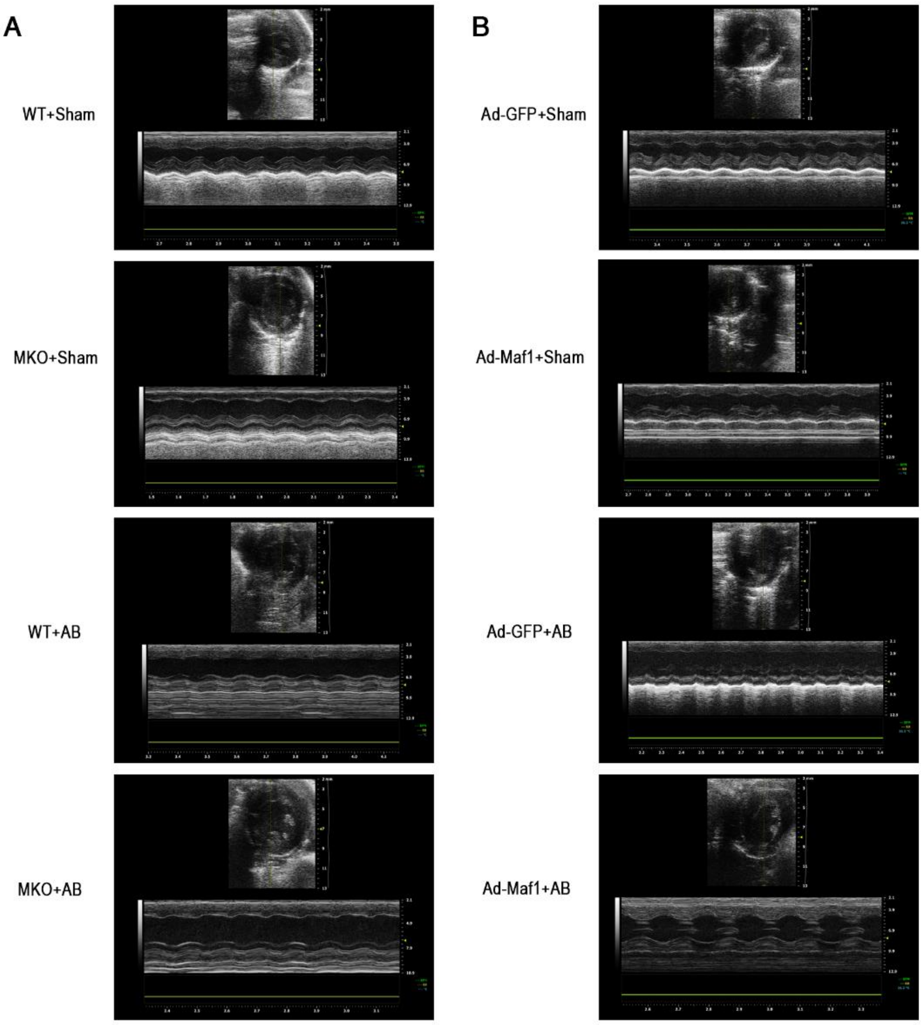

Supplement Figure 4. The M-mode echocardiogram image of mice. (A) Knockout of Maf1

induced changes as shown by the M-mode echocardiogram. (B) Upregulation of Maf1 induced changes as shown by the M-mode echocardiogram.

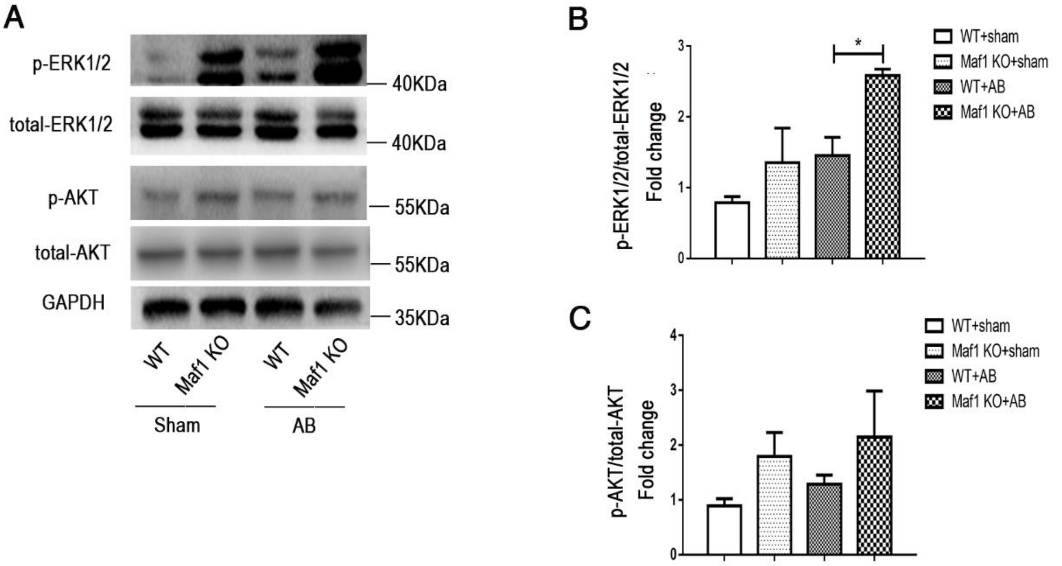

Supplement Figure 5. Effects of Maf1 deficiency on the ERK1/2 and AKT signaling pathways. The mice were subjected to AB surgery for 4 weeks. (A) Western blots showing the effect of Maf1 knockout on the ERK1/2 and AKT signaling pathways, and the quantitative analysis of those blots (B, C); n=4. *p<0.05 versus the corresponding control group.

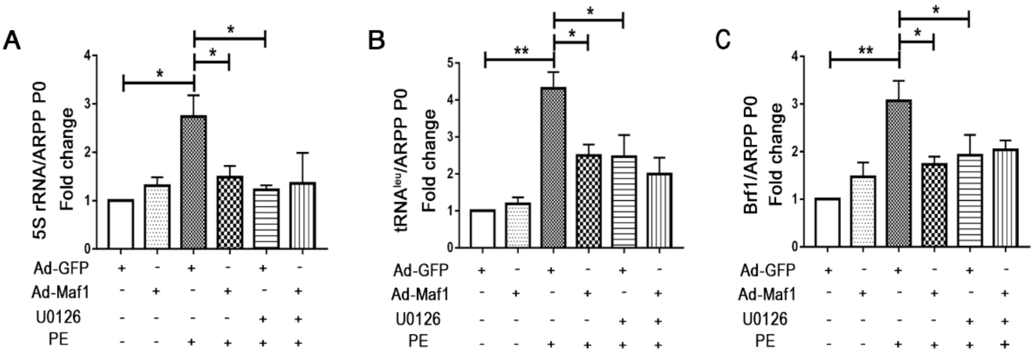

Supplement Figure 6. The effect of Maf1 upregulation and U0126 treatment on the relevant products of RNA pol III transcription. (A-C) The effect of Maf1 upregulation and U0126 treatment on expression levels of the relevant products of RNA pol III transcription 5SrRNA and $tRNA^{Leu}$ and the pol III subunit Brf1 were detected by q-PCR, and ARPP P0 was used as an internal control; n=4-7. *p<0.05 versus the corresponding control group. **p<0.01 versus the corresponding control group.

Supplement Methods

Maf1 knockout mouse construction

The procedure by which Maf1 knockout (Maf1 KO) mice (C57BL/6J background) were

established is described below. A transcription activator-like effector (TALE) nuclease targeted exon 3 of the Maf1 gene was designed using the targeter designer (*https://tale-nt.cac.cornell.edu/node/add/talen-old*). The sequences of the target sites were GCCTGCGTTCTTTAGGAT and ATGGCGGGAGATGATAAAC. TALEN plasmids were linearized and used as templates for *in vitro* transcription using the mMESSAGE mMACHINE T7 Ultra Kit (Ambion, Life Technologies), and the products were purified using the MEGAclearTM Kit (Ambion, Life Technologies) according to manufacturer's instructions. The TALEN mRNA was diluted to a working concentration of 50 ng/μl in injection buffer (10 mM Tris, 0.1 mM EDTA, pH 7.4) and injected into C57BL/6J zygotes by microinjection. The genomic DNA of F0 generation mice was extracted from the mouse tail, and an approximate 501-bp PCR product was obtained using the PCR sense primer 5'-GCCATGCTGGCTTCCCACAT-3' and antisense primer 5'-TTGTCGCTCAGAGGACTCTCATC -3'. The PCR products from F0 generation mouse tails were purified, cloned and sequenced to screen the positive founder mice for a Maf1 protein frame shift. The positive F0 generation mice were crossed with C57BL/6J mice. The genotypes of F0 offspring were further confirmed by PCR, cloning and sequencing.

In Vivo Myocardial Gene Transfer

The thoracotomy was performed in the left fifth intercostal space, the pericardium was opened. Adenoviruses (1×10^9 total particles, 10 μl) were diluted in 20 μl phosphate-buffered saline (PBS) and prepared for injection. Then, we used tweezers to clamp the aorta and pulmonary artery and the adenoviruses were injected into the left ventricular (LV) cavity. This procedure allows the solution that contains the adenovirus to circulate down the coronary arteries and perfuse the heart. After 5 seconds, the tweezers were loosened, and the chest was sutured. Theoretically, the adenoviruses entered the myocardium evenly through the coronary artery. The control group was injected with the same amount of Ad-GFP diluted in PBS [1-2].

Author contribution statement

In this research study, Yugang Dong and Chen Liu were responsible for the project design and data analysis. Yu Sun and Cong Chen were responsible for the major experiments, including neonatal rat ventricular cardiomyocyte culture, recombinant adenovirus and siRNA transfection, immunoprecipitation and immunoblot analyses. Ruicong Xue and Yan Wang were responsible for animal feeding and surgery. Yu Sun and Ruicong Xue were also responsible for the major supplementary experiment and writing and revision of the paper. Bin Dong and Jingzhou Jiang were responsible for the cytoplasmic and nuclear protein extraction. Wendong Fan was responsible for the measurement of cell surface area. Zhuomin Liang and Huiling Huang were responsible for the immunofluorescence staining. Rong Fang and Gang Dai were responsible for the real-time quantitative PCR and reverse transcription. Jiayong Li, Chen Chen, Youchen Yan, Tiqun Yang, Zhan-Peng Huang and Xiangxue Li were participated in the supplementary experiment.

Reference

1. Roth DM, Lai NC, Gao MH, Drumm JD, Jimenez J, Feramisco JR, et al. Indirect

intracoronary delivery of adenovirus encoding adenylyl cyclase increases left ventricular contractile function in mice. American Journal of Physiology-Heart and Circulatory Physiology. 2004; 287: H172-H177.
2. Qin Y, Yu Y, Dong H, Bian X, Guo X, Dong S. MicroRNA 21 inhibits left ventricular remodeling in the early phase of rat model with ischemia-reperfusion injury by suppressing cell apoptosis. International journal of medical sciences. 2012; 9: 413.
